# Supplementary material for: Maintaining extensivity in evolutionary multiplex networks
Source: PLoS One. 2017 Apr 12;12(4):e0175389. doi: 10.1371/journal.pone.0175389 (PMC5389798; doi:10.1371/journal.pone.0175389)
Supplement: S1 File — (PDF) [file pone.0175389.s001.pdf]

Maintaining extensivity in evolutionary multiplex networks

Supporting Information S1

Chris G. Antonopoulos[1] and Murilo S. Baptista[2]

[1]Department of Mathematical Sciences, University of Essex, Wivenhoe Park, UK
[2]Institute of Complex Sciences and Mathematical Biology, University of Aberdeen, SUPA, Aberdeen, UK

Abstract

In this Supporting Information, using our analytical derivations from the networks of shift maps considered in the paper, we first demonstrate that for a single network, extensivity of the sum of the positive Lyapunov exponents, H_{KS}, typically implies that the probability density of Lyapunov spectrum collapses to a universal curve, which in turn implies its invariance. Our second demonstration is that, in contrast to the former class of systems, extensivity can also be typically observed in our studied multiplex networks, however depending on how topology and coupling strengths are altered as the network grows, the existence of an invariant Lyapunov spectrum may not be observed.

Extensivity versus scaling of Lyapunov spectra

Let us consider the probability density of the Lyapunov exponents (LEs) λ_i, $\rho(\lambda_i)$. If $\rho(\lambda_i)$ is invariant for sufficiently large N, then the distribution $\rho(\omega_i)$ of the eigenvalues will also be invariant, since the LEs are just the logarithm of a constant minus the weighted eigenvalues. Invariance of the probability distribution of the LEs implies invariance of the Lyapunov spectrum. Thus, here we will study the properties of the distribution of the eigenvalues, i.e. of $\rho(\omega_i)$, and based on this, we will then explain the properties of the Lyapunov spectrum, namely of the set of the ordered LEs.

Demonstration 1: Extensivity when the network topology is preserved as the network evolves

In the following, we proceed with calculations considering single networks studied in previous works [1, 2], i.e. only taking into consideration either of the subnetworks G_1 or G_2.

Assuming that all LEs of such a subnetwork are positive, their sum is given by

$$H_{KS} \approxeq N_1 \log(2) - \frac{\epsilon(N_1)}{2} S, \tag{1}$$

where

$$\sum_{i=1}^{N_1} \omega_i = \sum_{i=1}^{N_1} d_i \equiv S, \tag{2}$$

d_i is the intra-degree of node i in G_1 or G_2 and N_1 is the number of nodes in G_1 or G_2.

The normalised eigenvalues can be written as

$$\omega_i' = \frac{\omega_i}{\omega_{N_1}},$$

and therefore,

$$\sum_{i=1}^{N_1} \omega_i' = \frac{1}{\omega_{N_1}} \sum_{i=1}^{N_1} \omega_i. \tag{3}$$

In the limit of $N_1 \to \infty$, we can write

$$\frac{1}{N_1}\sum_{i=1}^{N_1}\omega_i = \int_0^{\omega_{N_1}} \rho(\omega)\omega d\omega, \tag{4}$$

$$\frac{1}{N_1}\sum_{i=1}^{N_1}\omega_i' = \int_0^{1} \rho'(\omega')\omega' d\omega', \tag{5}$$

where Eq. (5) confines the probability density into $[0, 1]$, regardless of the size N_1 of the subnetwork, and allow us to compare the form of the distribution for different network sizes.

To understand the connection between the invariance of Eqs. (4) and (5) with respect to N_1, and the invariance of the curve of the ranked Laplacian matrix eigenvalues, and consequently the invariance of the Lyapunov spectrum, we proceed to an example, based on a star topology. For such a network, one has a Laplacian with the set of eigenvalues $\{\omega_1 = 0, \omega_k = 1, \omega_{N_1}\}$, where $k = 2, \ldots, N_1 - 1$. As N_1 goes to infinity, the probability distribution of the eigenvalues approaches a delta function at $\omega = 1$, with one singular point at infinity and another one at zero. In the limit of $N_1 \to \infty$, this set has an average value for the eigenvalues which is equal to 2. The curve for the eigenvalues, ordered by the index $h = k/N_1$, asymptotically approaches the line $\omega(h) = 1$, neglecting the singular points $\omega(h = 0) = 0$ and $\omega(h = 1) = N_1$. The Lyapunov spectrum associated with these eigenvalues approach the line $\log(2 - \epsilon(N_1))$, neglecting the extremal points. The normalised distribution in Eq. (5) approaches a delta function at $\omega' = 0$, which is invariant with respect to N_1, producing an average normalised eigenvalue equal to 0.

For the subnetwork to be extensive, $\frac{\epsilon(N_1)}{2}S$ must be a linear function of N. Replacing S by the quantities in Eq. (2), and enforcing an extensive (linear with N_1) behaviour in $H_K S$ in Eq. (1), we have that

$$\frac{\epsilon(N_1)}{2}S \equiv \frac{\epsilon(N_1)}{2}\sum_{i=1}^{N_1} d_i \equiv \frac{\epsilon(N_1)}{2}\sum_{i=1}^{N_1}\omega_i = \sigma N_1. \tag{6}$$

If $\rho(\omega')$, in the limit of $N_1 \to \infty$, is an invariant curve irrespective of N_1, Eq. (5) is invariant with respect to N_1, and is equal to a constant value for any sufficiently large N_1. Let us make a general assumption that the average calculated by Eq. (5) is a function represented by $C(\Lambda, N_1)$ of some characteristic of the network (e.g. the average degree, $\bar{d}$) which we denote by Λ, with N_1 nodes, i.e. $C(\Lambda, N_1) = \frac{1}{N_1}\sum_{i=1}^{N_1}\omega_i'$.

From Eqs. (3) and (6), we have that $\sum_{i=1}^{N_1}\omega_i' = 2\frac{\sigma N_1}{\omega_{N_1}\epsilon(N_1)}$. Substituting this into the left-hand side of Eq. (5), to maintain extensivity, we require that

$$\frac{2\sigma}{\omega_{N_1}\epsilon(N_1)} = C(\Lambda, N_1). \tag{7}$$

For an invariant curve for the Lyapunov spectrum, we require that $\rho(\omega)$ and $\rho(\omega')$ are invariant distributions, irrespectively of the network size and characteristics. Thus, $C(\Lambda, N_1) = C$ must be a constant.

Then, all that is required for an extensive subnetwork to also have an invariant Lyapunov spectrum is that the left-hand side of Eq. (7) is constant, which leads to

$$\omega_{N_1}\epsilon(N_1) = C_1, \tag{8}$$

where C_1 is a constant.

Therefore, if we choose

$$\epsilon(N_1) = \frac{C_1}{\omega_{N_1}},$$

to maintain extensivity, we will also set $C(\Lambda, N_1) = \frac{2\sigma}{C_1}$ to be a constant, implying the invariance of the curve for the Lyapunov spectrum.

Thus, as the network grows, there will be an infinite number of choices the diffusive coupling $\epsilon(N_1)$ can assume that lead to extensivity, even for finitely-sized networks. However, C_1 must be

adjusted in order for the network to only have positive LEs (thus $\epsilon(N_1)\omega_i < 1$), otherwise, Eq. (1) is not valid. For example, in the case of an all-to-all network, $\omega_{N_1} = N_1$ and $C_1 < 1$. In this case, the Lyapunov spectrum will collapse to the value $\log(2 - C_1)$, generating an invariant line for the curve of the Lyapunov spectrum. For any other network, the LEs can be calculated by $\log(2 - \epsilon(N_1)\omega_i)$=$\log(2 - C_1\omega_i')$. Any network, and there are several ones [3], with a spectrum of eigenvalues following an asymptotic probability density, will produce an invariant Lyapunov spectrum in the limit of $N_1 \to \infty$.

Demonstration 2: Extensivity when the multiplex network topology is changed in order to maximise H_{KS}

We now study how topology is related to extensivity, when inter-links are modified (in the strength and topology) between the subnetworks G_1 and G_2 to maximise the sum of LEs given by

$$H_{KS} \approxeq N\log(2) - \epsilon(N_1)S - \gamma l_{12},$$

where l_{12} represents the number of inter-connections between G_1 and G_2.

Extensivity in the multiplex network is achieved if H_{KS} is a linear function of N, and this leads to the requirement that $\epsilon(N_1)S + \gamma l_{12} = \sigma N$, which using Eq. (2) can be written as

$$\epsilon(N_1)\sum_{i=1}^{N_1}\omega_i + \gamma l_{12} = \sigma N + \nu, \tag{9}$$

where ν is a constant.

In this case, it is interesting to study the set of the weighted eigenvalues of the multiplex network (which takes into consideration the coupling strengths), given by

$$\begin{aligned}\mu_{2i-1} &= \epsilon(N_1)\omega_i,\\ \mu_{2i} &= \epsilon(N_1)\omega_i + 2\gamma\alpha,\end{aligned} \tag{10}$$

with $i = \{1, \ldots, N_1\}$ and $\alpha = \frac{l_{12}}{N_1}$. Let us denote the probability densities of μ_{2i-1} and μ_{2i} by $\rho(\mu_{2i-1})$ and $\rho(\mu_{2i})$, respectively. We then define the normalised weighted eigenvalues

$$\mu'_{2i-1} = \frac{\epsilon(N_1)\omega_i}{\mu_{2N_1-1}},$$

and therefore,

$$\sum_{i=1}^{N_1}\mu'_{2i-1} = \frac{1}{\mu_{2N_1-1}}\sum_{i=1}^{N_1}\epsilon(N_1)\omega_i. \tag{11}$$

In the limit of $N_1 \to \infty$, we have that

$$\frac{1}{N_1}\sum_{i=1}^{N_1}\mu_{2i-1} = \int_{\mu_1}^{\mu_{2N_1-1}}\rho(\mu_{2i-1})\mu_{2i-1}d\mu_{2i-1},$$

$$\frac{1}{N_1}\sum_{i=1}^{N_1}\mu'_{2i-1} = \int_0^1\rho'(\mu'_{2i-1})\mu'_{2i-1}d\mu'_{2i-1}, \tag{12}$$

$$\frac{1}{N_1}\sum_{i=1}^{N_1}\mu_{2i} = \int_{\mu_2}^{\mu_{2N_1}}\rho(\mu_{2i})\mu_{2i}d\mu_{2i}. \tag{13}$$

As we grow the network (i.e. as N_1 is increasing), the multiplex network topology evolves to some characteristics denoted by Λ, such as the average degree $\bar{d}$. We can write that $\frac{1}{N_1}\sum_{i=1}^{N_1}\mu'_{2i-1} = C(\Lambda, N_1)$, which substituting in Eq. (11) provides us with $\epsilon(N_1)\sum_{i=1}^{N_1}\omega_i = C(\Lambda, N_1)N_1\mu_{2N_1-1}$. Plugging this result into the extensivity constrain in Eq. (9), we get

$$C(\Lambda, N_1)\frac{N}{2}\mu_{2N_1-1} + \gamma l_{12} = \sigma N + \nu. \tag{14}$$

It is also useful to know that

$$C(\Lambda, N_1) = \frac{\epsilon(N_1)\sum_{i=1}^{N_1}\omega_i}{N_1\mu_{2N_1-1}} = \frac{\epsilon(N_1)\bar{d}}{\mu_{2N_1-1}}. \tag{15}$$

If Eq. (12) is invariant with respect to N_1, then $C(\Lambda, N_1) = C$ is constant.

At each step of the evolution (for each multiplex network of subnetworks with N_1 nodes), an optimisation process chooses an optimal set of inter-links and coupling strengths γ that maximise H_{KS}, assuming both $|\gamma| > 0$ and maximisation of the LEs in Eq. (10) can be accomplished by maximising $\log|2-\mu_{2i}|$, still maintaining all LEs positive. This can be accomplished by solving $|2-\mu_{2N_1}| = 2$, since $\mu_i \leq \mu_{i+1}$. However, doing this does not respect the choice made in the paper that all our theoretical and numerical calculations have considered positive coupling strengths. Thus, we proceed in the following assuming that the coupling strengths are positive. We moreover assume that the optimisation process calculates different network topologies and inter-coupling strengths to maintain the positiveness of the LEs, leading to $\log|2-\mu_{2i}| > 0$, which implies $2 - \epsilon(N_1)\omega_i - 2\gamma\alpha > 1$ and $\epsilon(N_1)\omega_i < 1$ for all i (remind that ω_1=0). This results in

$$\gamma l_{12} < \frac{N}{4}(1 - \epsilon(N_1)\omega_{N_1}), \tag{16}$$

$$\epsilon(N_1)\omega_{N_1} < 1. \tag{17}$$

Equations (16) and (17) can be transformed into an equality by the use of a constant parameter C_1, leading to $\epsilon(N_1) = \frac{C_1(\Lambda,N_1)}{\omega_{N_1}}$ with $C_1(\Lambda, N_1) < 1$,

$$\gamma l_{12} = \frac{N}{4}(1 - C_1(\Lambda, N_1)), \tag{18}$$

$$\epsilon(N_1)\omega_{N_1} = C_1(\Lambda, N_1). \tag{19}$$

Notice that Eq. (18) implies that $2\gamma\alpha = (1 - C_1(\Lambda, N_1))$. Since $\mu_{2N_1-1} = \epsilon(N_1)\omega_{N_1}$, we obtain using Eq. (19) that

$$\mu_{2N_1-1} = C_1(\Lambda, N_1) = \epsilon(N_1)\omega_{N_1}. \tag{20}$$

Equation (15) can be rewritten as

$$C(\Lambda, N_1) = \frac{\bar{d}}{\omega_{N_1}}. \tag{21}$$

Since $\mu_{2N_1} = \mu_{2N_1-1} + 2\gamma\alpha$, then

$$\mu_{2N_1} = 1.$$

Thus, all eigenvalues of the Laplacian matrix of the multiplex network are confined within $[0, 1]$.

Substituting Eqs. (18) and (20) to Eq. (14) leads to

$$C(\Lambda_N, N_1)C_1(\Lambda_N, N_1)\frac{N}{2} + \frac{N}{4}(1 - C_1(\Lambda_N, N_1)) = \sigma N + \nu. \tag{22}$$

There are different ways to achieve extensivity. For example, it can be maintained if $\sigma = \frac{1}{4}(1 - C_1(\Lambda_N, N_1)) + \frac{C(\Lambda_N,N_1)C_1(\Lambda_N,N_1)}{2}$ is constant (which implies both $C(\Lambda_N, N_1)$ and $C_1(\Lambda_N, N_1)$ be constant) and $\nu = 0$.

Using the values of $C(\Lambda, N_1)$ and $C_1(\Lambda, N_1)$, from Eqs. (21) and (19), respectively, we obtain that

$$C(\Lambda, N_1)C_1(\Lambda, N_1) = \epsilon(N_1)\bar{d}. \tag{23}$$

Therefore, using Eqs. (23) and (19), Eq. (22) can be rewritten as

$$\epsilon(N_1)\bar{d}\frac{N}{2} + \frac{N}{4}(1 - \epsilon(N_1)\omega_{N_1}) = \sigma N + \nu. \tag{24}$$

Notice however that in order to obtain an invariant Lyapunov spectrum for the dynamics in the multiplex network, it is required that not only Eq. (12), but also Eq. (13) is invariant, and that $\frac{1}{N_1}\sum_{i=1}^{N_1}\mu_{2i}$ is constant for any N_1. We would expect that

$$\frac{1}{N_1}\sum_{i=1}^{N_1}(\epsilon(N_1)\omega_i + 2\gamma\alpha) = C_2,$$

with C_2 being a constant. Then, the problem reduces to demonstrating that

$$\frac{1}{N_1}\sum_{i=1}^{N_1}(\epsilon(N_1)\omega_i + 1 - C_1(\Lambda, N_1)) = C_2. \tag{25}$$

Calculating $\epsilon(N_1)$ from Eq. (19) to optimise H_{KS} and to maintain extensivity, and placing it in Eq. (25), we obtain that

$$\frac{1}{N_1}\sum_{i=1}^{N_1}\left(C_1(\Lambda, N_1)\frac{\omega_i}{\omega_{N_1}} + 1 - C_1(\Lambda, N_1)\right) = C_1(\Lambda, N_1)\left(\frac{\bar{d}}{\omega_{N_1}} - 1\right) + 1 =$$
$$C_1(\Lambda, N_1)[C(\Lambda, N_1) - 1] + 1 = C_2. \tag{26}$$

Let us now analyse 2 cases:
Case 1: Assume that $C_1(\Lambda, N_1) = C_1 < 1$ is constant. From Eq. (19), this choice implies that $\epsilon(N_1) = C_1/\omega_{N_1}$, if all LEs are to be maintained positive by the optimisation process. To maintain extensivity (see Eq. (22)) $C(\Lambda, N_1) = C$ is also chosen to be constant. Then, from Eq. (26), we conclude that $C_2(\Lambda, N_1)$ is also a constant. These types of networks will not only be extensive, but may also possess an invariant Lyapunov spectrum. For example, for circulant networks where each node has a degree k, and a largest eigenvalue ω_{N_1} that scales with $2k$, we have that

$$\lim_{N_1\to\infty}\frac{\bar{d}}{\omega_{N_1}} \equiv C(\Lambda, N_1) \propto \frac{1}{2}.$$

Case 2: Here we analyse 3 network characteristics:
Case 2.1: Let us choose $C_1(\Lambda, N_1) \propto \frac{\omega_{N_1}}{N\bar{d}}$, which implies $\epsilon(N_1) \propto \frac{1}{N\bar{d}}$, such that

$$C(\Lambda, N_1)C_1(\Lambda, N_1) \propto \frac{1}{N}.$$

Then, Eq. (22) can be rewritten as

$$\frac{1}{2} + \frac{N}{4} - \frac{\omega_{N_1}}{4\bar{d}} = \sigma N + \nu. \tag{27}$$

If $C(\Lambda, N_1) = \frac{\bar{d}}{\omega_{N_1}}$ is constant as the network grows, the network will be extensive with $\sigma = \frac{1}{4}$ and $\mu = 1/2 - C_1$ (see Eq. (27)), and will have an invariant curve for the Lyapunov spectrum, since both $C(\Lambda, N_1)$ and $C_2(\Lambda, N_1)$ will be constants and the probability densities of the weighted eigenvalues will be invariant with respect to N_1.
Case 2.2: Let us choose $\epsilon(N_1) \propto \frac{1}{N\bar{d}}$, e.g. $\epsilon(N_1) = \alpha\frac{1}{N\bar{d}}$. If $\frac{\omega_{N_1}}{\bar{d}}$ scales as N, i.e. $\frac{\omega_{N_1}}{\bar{d}} = \beta N + \xi$, then putting this into Eq. (27), one sees that the multiplex network will still be extensive with a extensivity coefficient equal to $\sigma = \frac{1}{4} - \frac{\alpha\beta}{4}$, however $C(\Lambda, N_1) = (\beta N + \xi)^{-1}$ will not be constant, and thus the probability distributions of the weighted eigenvalues will not be invariant as N_1 increases. Consequently, as the network grows the curve for the Lyapunov spectrum will not approach an invariant curve. The half set of LEs in the thermodynamic limit (based on μ_{2i}) is given by $\lambda_{2i} = \log\left(2 - \alpha\beta\left(\frac{\omega_i}{\omega_{N_1}} - 1\right) - 1\right)$, and the other half (based on μ_{2i-1}) is given by $\lambda_{2i-1} = \log\left(2 - \alpha\beta\frac{\omega_i}{\omega_{N_1}}\right)$. If $\alpha = \beta = 1$, then $\lambda_{2i} = \log\left(2 - \frac{\omega_i}{\omega_{N_1}}\right)$ and $\lambda_{2i-1} = \log\left(2 - \frac{\omega_i}{\omega_{N_1}}\right)$, thus the LEs will be degenerated, and the Lyapunov spectrum will not be invariant, since $\omega_{N_1} \propto \bar{d}N$. Here, it becomes clear that the density of the ratio (ω_i/ω_{N_1}) is crucial for the invariance (or non-invariance) of the curve for the Lyapunov spectrum. Derivation of the LEs can be done by using the fact that $\mu_{2i} = \epsilon\omega_i + (1 - C_1) = \epsilon(\omega_i - \omega_{N_1}) + 1$, then finally substituting the chosen function value for ϵ. For finite N, the Lyapunov spectrum receives a significant contribution from the constant term ξ, resulting in an apparent non-invariant curve for the LEs.
Case 2.3: From Eq. (19), let us choose $C_1(\Lambda, N_1) = \frac{\omega_{N_1}}{N}$ such that $\epsilon(N_1) = \frac{1}{N}$ and increase the average degree, $\bar{d}$, linearly with N as the network grows, i.e. $\bar{d} = \alpha N + \xi$. Equation (24) can be rewritten as

$$\frac{N}{4} - \frac{\omega_{N_1}}{4} + \frac{\alpha N + \xi}{2} = \sigma N + \nu,$$

and the network will be extensive with $\sigma = \frac{1}{4} - \frac{\omega_{N_1}}{4N} + \frac{\alpha}{2}$. Both $C(\Lambda, N_1) = \frac{\alpha N + \xi}{\omega_{N_1}}$ and $C_2(\Lambda, N_1)$ will not be constant, and therefore, there will be no invariant density of weighted eigenvalues, and consequently no invariant Lyapunov spectrum. The set of LEs in the limit of $N_1 \to \infty$ are given by $\lambda_{2i} = \log\left(2 - \frac{1}{N}(\omega_i - \omega_{N_1}) - 1\right)$, i.e. the set of LEs that produce the non-invariant Lyapunov spectrum, and $\lambda_{2i-1} = \log\left(2 - \frac{\omega_i}{N}\right)$. Here, it becomes clear that the density of the difference $(\omega_i - \omega_{N_1})$ is crucial in determining the invariance (or not) of the curve for the Lyapunov spectrum. For a finite N, the spectrum of LEs receives a significant contribution from the constant term ξ, resulting in an apparent non-invariant curve for the Lyapunov spectrum.

To illustrate **Cases 2.1 and 2.3**, let us study an Erdős−Rényi random network, with known degrees. The largest eigenvalue ω_{N1} is estimated by [3]

$$\omega_{N_1} \approxeq \frac{2}{\sqrt{\bar{d}}}.$$

For this network, $C(\Lambda, N_1) \approxeq \frac{\bar{d}^{3/2}}{2}$. If $\bar{d}$ remains constant as we grow the multiplex network (by increasing N_1), then the network will not only be extensive (with $\sigma = 1/4$), but will also have an invariant curve for the Lyapunov spectrum. In the limit of $N_1 \to \infty$, invariance of the density of weighted eigenvalues and Lyapunov curve is also naturally achieved (see **Case 2.1**). However, if we grow the network by increasing the degrees as we increase N_1, in order to make $\bar{d}(\Lambda, N_1) = \alpha N$, extensivity will be maintained by choosing $\epsilon(N_1) \propto \frac{1}{N}$, with $\sigma = \frac{1}{4} + \frac{\alpha}{2}$ (for $\epsilon(N_1) = 1/N$). However, both $C \propto \frac{N}{\omega_{N_1}}$ and C_2 will not be constants, and therefore, there will be no invariant density of weighted eigenvalues, and consequently the curve for the Lyapunov spectrum will not be invariant (see **Case 2.2**).

Conclusion about extensivity versus invariance of the Lyapunov spectra

Concluding, while single subnetworks constructed to be extensive typically produce invariant curves for the Lyapunov spectra, multiplex networks can be expected to be extensive possibly giving rise to non-invariant curves for the Lyapunov spectra. This may happen for example, when the networks grow by altering either their topological characteristics or their coupling strengths.

Characteristics of evolved networks of Hindmarsh-Rose neurons

In the following, we define $d_i^{G_1}$ and $d_i^{G_2}$ to be the intra-degrees of the networks of the initial, non-evolved subnetworks G_1 and G_2, respectively. We then define

$$S^{G_1} = \sum_{1}^{N_1} d_i^{G_1},$$

$$S^{G_2} = \sum_{1}^{N_1} d_i^{G_2},$$

and

$$S^{\text{intra}} = S^{G_1} + S^{G_2}.$$

The average intra-degree $\bar{d}$ of the non-evolved initial networks can then be calculated as

$$\bar{d} = \frac{S^{\text{intra}}}{N}.$$

From the data in Fig. 1, we obtain performing a linear fitting that

$$\bar{d} = 3.3333(\pm 0.3212) + 0.0179(\pm 0.0103)N,$$

$$\frac{\omega_{N_1}}{\bar{d}} = 1.8626(\pm 0.1002) - 0.0007(\pm 0.0032)N,$$

N	$\omega_{N_1}^{G_1}$	$\omega_{N_1}^{G_2}$
08	5.6457	4.000
16	6.8190	6.5792
24	7.9783	6.9198
32	7.3372	6.9043
40	7.3952	7.2668
48	7.1424	7.1016

Table 1: **The largest eigenvalue ω_{N_1} of subnetworks G_1 and G_2, considering only their intra-topologies.** The first column is the number of nodes N in the non-evolved network, whereas the second and third columns are the largest eigenvalues ω_{N_1} of subnetworks G_1 and G_2, respectively.

where the numbers in the parentheses denote the asymptotic standard error in the parameter computations.

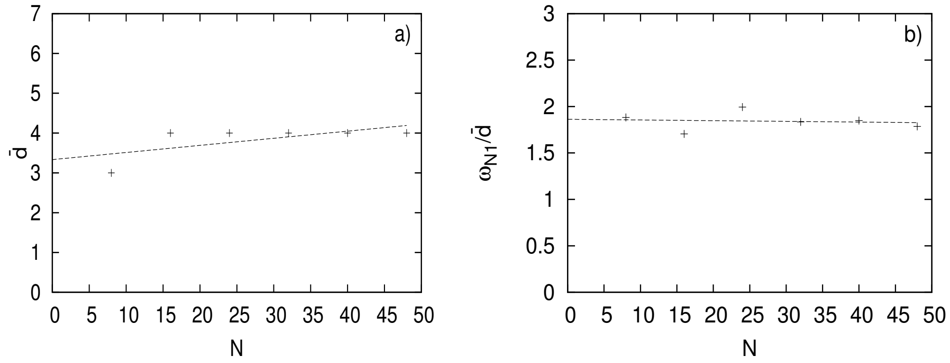

Figure 1: **Analysis of the characteristics of non-evolved subnetworks.** In panel a) we show $\bar{d}$ vs N and in panel b) the quantity $\frac{\omega_{N_1}}{\bar{d}}$ vs N.

In Table 1, we show in the first column the number of nodes N in the non-evolved multiplex network and, in the second and third columns the largest eigenvalue ω_{N_1} of subnetworks G_1 and G_2, respectively, considering only their intra-topologies.

References

[1] Ruelle, D. Large volume limit of the distribution of characteristic exponents in turbulence. *Communications in Mathematical Physics* **87**, 287–302 (1982).

[2] Livi, R and Politi, A and Ruffo, S Distribution of characteristic exponents in the thermodynamic limit. **19**, 2033–2040 (1986).

[3] Chung, F and Linyuan, L and VuLivi, V Spectra of random graphs with given expected degrees. **100**, 6313–6318 (2003).
